# Mesenchymal Stem Cell Therapy in Acute Intracerebral Hemorrhage: A Dose-Escalation Safety and Tolerability Trial

**DOI:** 10.1007/s12028-023-01897-w

**Published:** 2023-12-19

**Authors:** Nisha C. Durand, H. G. Kim, Vishal N. Patel, Marion T. Turnbull, Jason L. Siegel, David O. Hodge, Rabih G. Tawk, James F. Meschia, W. David Freeman, Abba C. Zubair

**Affiliations:** 1 Center for Regenerative Biotherapeutics, Mayo Clinic, 4500 San Pablo Road, Jacksonville, FL 32224 USA; 2 Human Cellular Therapy Laboratory, Mayo Clinic, Jacksonville, FL USA; 3 Clinical Research Intern Scholar Program, Mayo Clinic, Jacksonville, FL USA; 4 Division of Neuroradiology, Mayo Clinic, Jacksonville, FL USA; 5 Research Collaborator in the Department of Neurology, Mayo Clinic, Jacksonville, FL USA; 6 Department of Critical Care Medicine, Mayo Clinic, Jacksonville, FL USA; 7 Biostatistics Unit, Mayo Clinic, Jacksonville, FL USA; 8 Department of Neurologic Surgery, Mayo Clinic, Jacksonville, FL USA; 9 Department of Neurology, Mayo Clinic, Jacksonville, FL USA; 10 Department of Laboratory Medicine and Pathology, Center for Regenerative Biotherapeutics, Mayo Clinic, Jacksonville, FL USA

**Keywords:** Acute ICH, Mesenchymal stem/stromal cells, Immunomodulation, Feasibility, Biomarkers, Safety

## Abstract

**Background:**

We conducted a preliminary phase I, dose-escalating, safety, and tolerability trial in the population of patients with acute intracerebral hemorrhage (ICH) by using human allogeneic bone marrow–derived mesenchymal stem/stromal cells.

**Methods:**

Eligibility criteria included nontraumatic supratentorial hematoma less than 60 mL and Glasgow Coma Scale score greater than 5. All patients were monitored in the neurosciences intensive care unit for safety and tolerability of mesenchymal stem/stromal cell infusion and adverse events. We also explored the use of cytokines as biomarkers to assess responsiveness to the cell therapy. We screened 140 patients, enrolling 9 who met eligibility criteria into three dose groups: 0.5 million cells/kg, 1 million cells/kg, and 2 million cells/kg.

**Results:**

Intravenous administration of allogeneic bone marrow–derived mesenchymal stem/stromal cells to treat patients with acute ICH is feasible and safe.

**Conclusions:**

Future larger randomized, placebo-controlled ICH studies are necessary to validate this study and establish the effectiveness of this therapeutic approach in the treatment of patients with ICH.

**Supplementary Information:**

The online version contains supplementary material available at 10.1007/s12028-023-01897-w.

## Introduction

Intracerebral hemorrhage (ICH) is a common and disproportionately deadly stroke subtype characterized by bleeding into the brain parenchyma and potentially the ventricles [1–3]. ICH accounts for only about 10–15% of all strokes but is one of the most lethal subtypes, with less than 50% of affected patients surviving by 1 year after stroke [4]. ICH also disproportionately affects Black and Asian American populations [3, 4]. Major risk factors for ICH include smoking, high alcohol consumption, and hypertension [5–8]. Neurologic deterioration is initially mediated by hematoma expansion and mass effect with the destruction of functional neuronal tissue and increase in intracranial pressure (ICP) [9]. In addition, edema, oxidative stress, neuroinflammation with systemic inflammation, localized microglia activation, and blood–brain barrier leakage are often present within the ICH site or surrounding tissue [10]. Early ICH intervention strategies include endotracheal intubation for airway protection and stabilization to prevent and possibly treat aspiration pneumonia [11], acute blood pressure control [12], anticoagulation reversal in those taking anticoagulants [2], ICP treatment, and, in select patients, possible neurosurgical evacuation of the hematoma or external ventricular drain (EVD) placement for cerebrospinal fluid diversion and ICP reduction.

Despite these existing ICH intervention strategies, mortality and morbidity after ICH remains high. US Food and Drug Administration–approved human trials of drugs and other experimental therapies, to date, have failed to yield a suitable candidate that improves ICH outcomes [1]. ICH triggers damage-associated molecular pattern HMGB-TLR4 pathway inflammation [13], inflammasome pathways of NLRC4 pyroptosis [14], and NLRP3-mediated neutrophil netosis [15]. Elevated neutrophil–lymphocyte ratio (NLR) in peripheral blood is increasingly observed with ICH [16], subarachnoid hemorrhage [17], and many other human diseases, including the coronavirus disease of 2019 (COVID-19) and colorectal cancer [18].

Mesenchymal stem/stromal cells (MSCs) have demonstrated several potential therapeutic benefits in ICH, including regeneration, immunomodulation, and antimicrobial activity, via paracrine-mediated signaling of cytokines, growth factors, extracellular vesicles, and antimicrobial peptides [19, 20]. MSCs also exhibit an immune-evasive phenotype [21] and can be isolated from multiple tissue types (e.g., bone marrow, adipose tissue, umbilical cord). Data obtained from preclinical animal models and human clinical studies have established that MSCs appear relatively safe and produce few adverse events (AEs) when delivered in vivo [22]. The efficacy of MSCs has been evaluated in multiple conditions, including heart failure, graft-versus-host disease, and Crohn’s fistula disease, with varying levels of effectiveness [23]. Patients with ICH may benefit from MSC therapy, as it has the potential to balance a deleterious inflammatory cascade, inhibit immune cell activation, and induce regeneration of damaged cells, neurons, and tissue at both the hematoma site and systemically. Administration of MSCs in preclinical ICH models have produced favorable results [10, 24]. Neurologic improvement in ICH rat models following administration of human bone marrow–derived MSCs (BM-MSCs) has been shown to be associated with synaptogenesis, neuronal migration, and reduced tissue loss [25, 26]. In addition, MSC delivery in preclinical ICH models attenuates inflammation in a manner that is dependent on the reduction of systemic proinflammatory cytokines such as interleukin 6 (IL-6) and interferon-γ [25].

The overall long-term goal of this study is to develop BM-MSC therapy for the treatment of acute ICH. The primary objective of this pilot study was to establish the safety and feasibility of administering allogeneic BM-MSCs to hospitalized patients with ICH within 7 days of onset. The safety of BM-MSC therapy was evaluated by assessing tolerance of the cell infusion and development of AEs. Feasibility was assessed by evaluating ease of recruitment and technical issues associated with cell preparation and infusion. Clinical parameters and biomarkers for future outcome assessment in larger studies using MSC therapy for recent ICHs were also identified.

## Methods

### Patient Population and Study Design

The study was a prospective dose-escalation, safety, and tolerability trial using MSC in human patients with ICH. Study patients were recruited from the inpatient services of the Mayo Clinic Comprehensive Stroke Center in Jacksonville, Florida. Inclusion criteria included patients aged 18 years or older with acute (< 168 h from onset) spontaneous supratentorial ICH, an ICH score of 1 to 4, hematoma volume less than 60 mL on admission via ABC/2 [27], and ability of patient or surrogate to provide written consent. Exclusion criteria included deep coma defined by a 5 or lower Glasgow Coma Scale (GCS) score, secondary ICH related to aneurysm, arteriovenous malformation, brain tumor or oral anticoagulants beside warfarin, active pregnancy, preexisting disability characterized by prestroke modified Rankin Scale (mRS) score greater than 2, history of malignancy within the last 5 years, evidence of significant liver or cardiac dysfunction as judged by the treating physician team, septicemia with fever and hemodynamic instability, or use of any experimental therapy within 3 months of study enrollment. A Consolidated Standards of Reporting Trials diagram and detailed eligibility criteria are listed in Supplemental Fig. 1 and Supplemental Table 1 [28]. This study was conducted under Investigational New Drug# 16510 from the US Food and Drug Administration, approved by the Mayo Clinic Institutional Review Board (Protocol # 15-003524) and registered at ClinicalTrials.gov (NCT03371329). Institutional review board–approved written informed consent was required from every participant or their legally authorized representative.

**Fig. 1:**
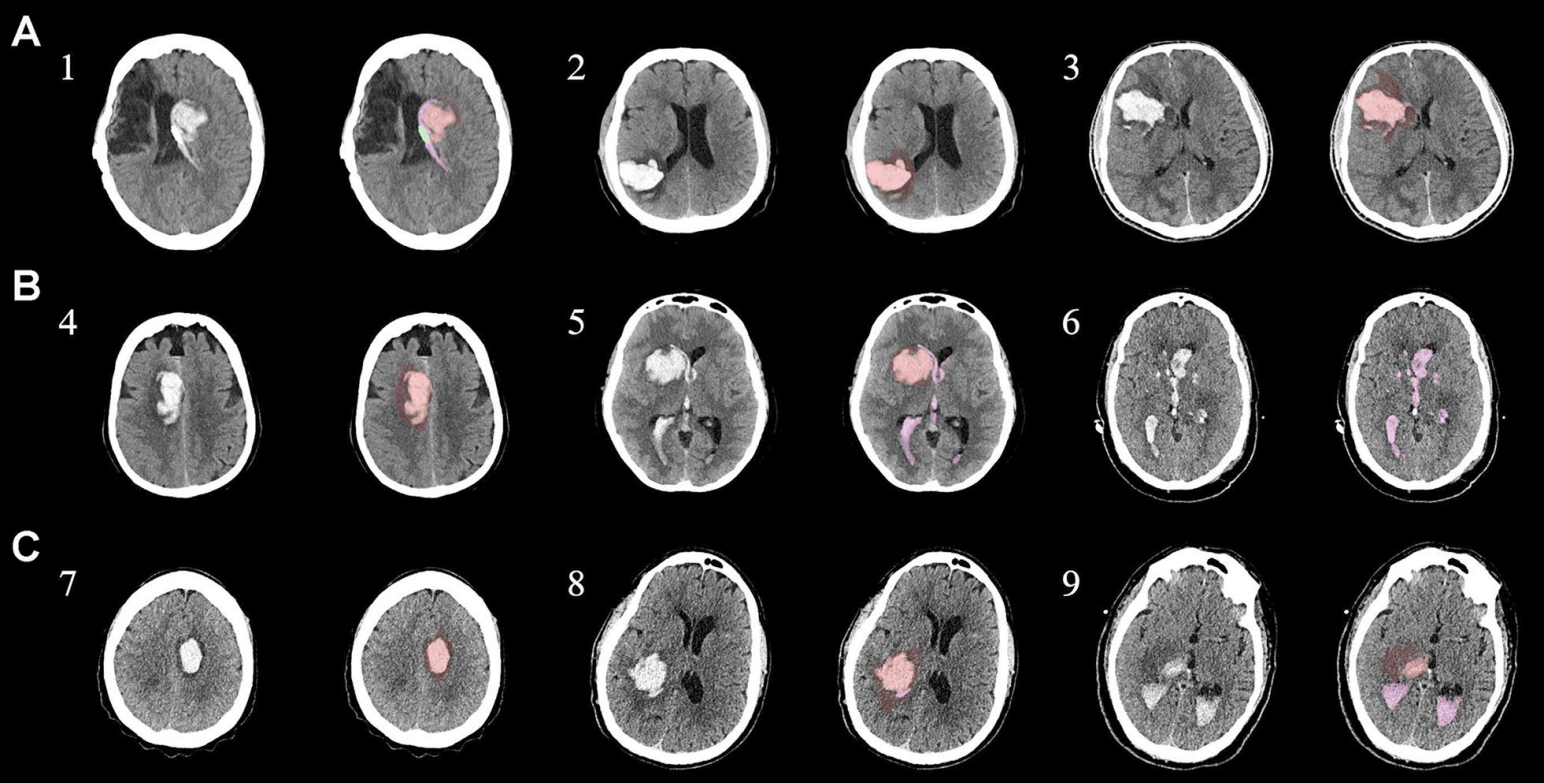
Original noncontrast computed tomography and segmentation results for each MSC-treated patient by group. **a**, Group 1, patients 1–3. **b**, Group 2, patients 4–6. **c**, Group 3, patients 7–9. The M1 ABC/2 [27] and modified Graeb measures used the hyperdense blood on the original images. Colors on the M2 segmentations represent intracerebral hemorrhage (orange), perihematomal edema (crimson), and intraventricular hemorrhage (pink). M1, model 1, M2, model 2, MSC, mesenchymal stem/stromal cell

**Table 1:** Patient characteristics and dose allocation

MSC group (10^6^ cells/kg)	Patient	Age	Sex	Admit NIHSS	Admit ICH score	Admit ICH vol M1	Admit ICH vol M2	Admit IVH vol M1	Admit IVH vol M2	Total blood vol M2	NLR	Discharge mRS
Group 1, 0.5	1	65	F	4	2	13.4	12.7	14	6.6	19.3	7.50	5
	2	60	M	8	2	53.1	54.9	3	4.6	59.5	NA	3
	3	65	M	4	1	45.7	44.1	0	0.0	44.1	7.90	2
Group 2, 1.0	4	84	F	2	2	26.3	26.4	10	9.7	36.1	14.28	4
	5	36	F	34	3	29.5	20.7	21	29	49.7	24.90	4
	6	47	F	35	3	0.24	0.1	29	60.9	61.0	8.80	5
Group 3, 2.0	7	51	F	13	1	18.9	13.3	1	0.3	13.6	17.96	0
	8	72	M	13	1	35.5	22.2	4	1.6	23.8	2.52	5
	9	72	M	25	2	21.2	17.4	17	33.6	51.0	8.56	5

F, female, ICH, intracerebral hemorrhage, IVH, intraventricular hemorrhage, M, male, mRS, modified Rankin Scale, M1, model 1, M2, model 2, MSC, mesenchymal stem/stromal cell, NA, not available, NIHSS, National Institutes of Health Stroke Scale, NLR, neutrophil–lymphocyte ratio, vol, volume

### MSC Manufacturing

Bone marrow–derived MSC manufacturing, processing, and delivery was performed by the Human Cellular Therapy Laboratory (HCTL) at the Mayo Clinic in Jacksonville, Florida, to the Mayo Clinic Hospital on the same campus [29]. The HCTL obtained bone marrow from a healthy donor who completed a comprehensive medical examination and institutionally approved Donor History Questionnaire. Infectious disease testing including human immunodeficiency virus 1 and 2 antibodies, human immunodeficiency virus nucleic acid testing (NAT), human T-lymphotropic virus type I and II antibodies, syphilis screening, hepatitis B surface antigen, hepatitis B core antibody, hepatitis B NAT, hepatitis C antibody, hepatitis C NAT, *Trypanosoma cruzi* antibody, West Nile virus NAT, Zika virus using enzyme-linked immunosorbent assay, and Zika using polymerase chain reaction was performed by a Clinical Laboratory Improvement–approved laboratory. After review of the medical evaluation of the donor, review of the negative infectious disease testing results and Donor History Questionnaire, donor eligibility determination was made by the HCTL Medical Director. After performing a bone marrow aspiration, we generated BM-MSCs from the approved donor by using manual methods of expansion.

MSC cultures were grown in Gibco Minimum Essential Medium α (Thermo Fisher Scientific) and supplemented with 5% Stemulate xeno-free and heparin-free pooled platelet lysate (Sexton Biotechnologies) along with 1X Gibco GlutaMAX supplement (Thermo Fisher Scientific). The final cell products were cryopreserved at 5 × 10^6^ MSCs/mL in CryoStor CS10 (10% dimethyl sulfoxide; STEMCELL Technologies Inc). The MSCs were stored in vapor phase liquid nitrogen at less than − 150 °C. We performed quality control testing on the cryopreserved cell product prior to release and patient delivery and infusion (Supplemental Table 2) [29].

**Table 2:** Summary of adverse events for enrolled patients

Patient	MSC group	Adverse event	Severity			Relation to study drug				
			Mild	Moderate	Severe	Unrelated	Unlikely related	Possibly related	Probably related	Definitely related
1	1	Fever		X				X		
		ESR/CRP abnormality	X				X			
		Hypertension		X			X			
		Change in cognitive status per NIHSS	X			X				
		Hgb decrease		X			X			
		UTI		X		X				
		Encephalopathy			X	X				
		Hydrocephalus			X	X				
3	1	Lower back pain			X	X				
		Bilateral leg pain			X	X				
4	2	Hypotension (vomiting and altered mental status)		X		X				
9	3	Cerebral hemorrhage and obstructive hydrocephalus			X	X				
		Wound infection			X	X				
		Seizure		X			X			
		Pneumonia		X		X				

CRP, C-reactive protein, ESR, erythrocyte sedimentation rate, Hgb, hemoglobin, MSC, mesenchymal stem/stromal cell, NIHSS, National Institutes of Health Stroke Scale, UTI, urinary tract infection

### MSC Preparation and Infusion

On the day of infusion, frozen BM-MSCs were thawed and diluted fivefold with PlasmaLyte A (Baxter) to yield a final concentration of 2.0% dimethyl sulfoxide. We infused 0.5 million to 2 million MSCs/kg intravenously, according to patient group. An aliquot of the final formulated product was reserved for quality control testing. We used 7-amino-actinomycin D to determine the number of viable cells present in the final formulated product. A minimum of 30,000 events were collected on a BD Accuri C6 Plus Flow Cytometer, and a gating strategy was used to separate the dead cells from the live cells. Gram staining and bacterial and fungal cultures were performed by Mayo Clinic’s Clinical Microbiology Lab.

MSC infusion was performed in the neurosciences intensive care unit; cells were infused at a rate of 2 to 3 mL/min during the first 15 min, with the option to be adjusted up to 5 mL/min if tolerated. Patients were monitored for AEs, and infusion toxicity was evaluated by monitoring the patient’s vital signs before, during, and up to 2 h after the MSC infusion. Patients were assigned to one of three dosing groups: group 1 received 0.5 million MSC/kg, group 2 received 1 million MSCs/kg, and group 3 received 2 million MSCs/kg (Table 1).

### Outcomes and Patient Monitoring

Safety was evaluated by assessing patients for their capacity to tolerate intravenous infusion without acute clinical or physiological deterioration. Additionally, AEs were coded using Medical Dictionary for Regulatory Activities standards [30] and categorized as mild, medium, or severe. Feasibility was evaluated by assessing the ability to recruit patients, determination of practical issues associated with cell product administration, and evaluation of patient compliance with study parameters. Laboratory testing, including complete blood count, liver function tests, and renal function tests were performed for all patients prior to MSC infusion (day 0) and on days 1, 2, 3, and 7 after infusion. Neurologic function tests including GCS, mRS [31], and the National Institutes of Health Stroke Scale (NIHSS) scores were evaluated on days 0, 1, 2, 3, 7, and 30. NIHSS was performed by either a neurologist or an experienced clinical research coordinator trained and certified in performing the scale.

### Biomarker Monitoring

Blood samples were collected on days 0 and 3 to evaluate quality and quantity of cytokines present by using a multiplex bead assay. Plasma was isolated from whole blood and stored at − 80 °C until cytokine analysis was performed. Plasma was analyzed by Eve Technologies for the presence and concentration of cytokines and growth factors using the Human Cytokine/Chemokine 65-Plex Discovery Assay Array (Eve Technologies Corp).

### Hematoma Volumetric Imaging by ABC/2 and Volumetric Segmentation

Given the importance of hemorrhage volume on prognosis [32], we graded the volume of ICH and intraventricular hemorrhage (IVH) by two methods we defined as model 1 and model 2. Model 1 used the ABC/2 method for estimating intracerebral (intraparenchymal) hematoma volume and the semiquantitative modified Graeb for IVH volume [33], which are both semiquantitative scales. For model 2, we used manually segmentation techniques for estimating both ICH and IVH volumes. We used the initial noncontrast computed tomography (CT) of the ICH or the noncontrast CT at hospital admission if there was hematoma expansion by 24 h. The ABC/2 method uses orthogonal linear measurements: A and B in the axial plane to capture the largest ICH cross-section and C, the vertical span, in increments of slice thickness. The halved product of these values ([A × B × C]/2) is a simplified formula that approximates ellipsoid hemorrhage. The ABC/2 is a well-known, easily implemented, and familiar method of ICH volume estimation. Model 2 or segmentation is a potentially more accurate method, but it is manually labor-intensive and requires dedicated volumetric segmentation software (RIL-Contour) [34]. However, segmentation of ICH and IVH volumetric methods are becoming increasingly automated [19, 35, 36] and benefit from machine and deep learning techniques such as convolutional neural networks. We also measured perihematomal edema (PHE) as an exploratory variable using segmentation methods around the hypodense areas on noncontrast CT relative to the ICH by manual segmentation. PHE was not measurable by ABC/2. ICH volume was measured by both methods in millimeters, and IVH volume measured by segmentation in millimeters were treated as continuous variables. ICH and IVH volume by ABC/2 and semiquantitative modified Graeb method for IVH measurements were established by a board-certified neuroradiologist (VNP), and segmentations were performed by a trained rater, followed by neuroradiologist validation. ABC/2 measurements were averaged among three trained raters (WDF, HKG, and VNP).

### Biostatistical Analysis

Descriptive and study population statistics were performed by using Microsoft Excel and SAS version 9.4. Linear regression models were performed by using GraphPad Prism 9.4.1, with confidence interval lines shown around the line of best fit by the *R*^*2*^ method.

## Results

### Patient Population

Patients with acute ICH within 168 h from onset from January 1, 2018, to October 31, 2020, were enrolled in this study. MSCs were intravenously administered to nine patients (five women, four men), with a mean (range) age of 61 (36–84) years. Demographic and clinical characteristics are summarized in Table 1. Patients were given numbers in order of enrollment for descriptive purposes and consecutively assigned to one of the three groups, three to each group. ICH volumes ranged from 0.1 to 54.9 mL by model 2 segmentation (mean ICH volume of 23.5 mL). Five ICHs had localized lobar involvement, with the rest being deep in either the basal ganglia or external capsule. In three patients, the ICH was localized in the thalamus (Fig. 1), and in one patient, the hematoma was localized to multiple brain structures (i.e., caudate, putamen, and globus pallidus). On average, MSCs were administered 3 days after ICH.

### MSC Characteristics

The mean (range) viability of the cell product prior to infusion was established as 78.2% (70.0–91.5%) by 7-amino-actinomycin D. No significant differences in viability were observed among the three groups. All post-thaw bacterial and fungal cultures were negative, and gram stain evaluations did not detect any organisms (Supplemental Table 2).

### Tolerance and Outcome of MSC Infusion

**Fig. 2:**
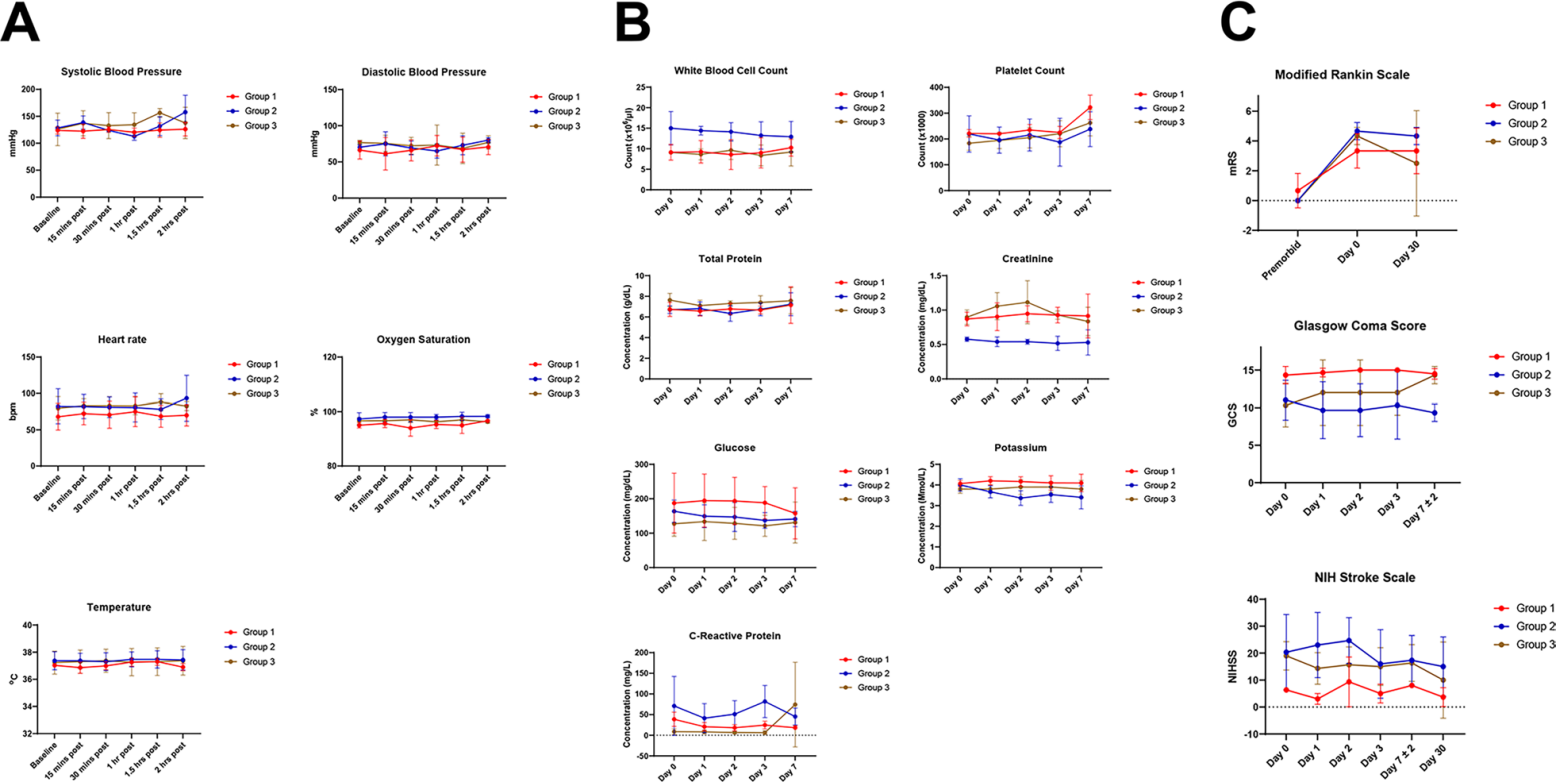
Safety and adverse event monitoring.** a**, Vitals measured at MSC infusion. **b**, Laboratory values measured before and after MSC infusion. **c,** Neurologic function test results before and after MSC infusion. MSC, mesenchymal stem/stromal cell

Patient vital signs remained stable following infusion. Compared with baseline values, systolic blood pressure, diastolic blood pressure, heart rate, oxygen saturation, and temperature measured 15 min, 30 min, 1 h, 1.5 h, and 2 h after infusion were not notably different (Fig. 2a). Figure 2b displays laboratory values measured on days 0, 1, 2, 3, and 7. White blood cell count, total protein, creatinine, and potassium were within normal physiological ranges, with no appreciable change between days 0 and 7. Throughout the evaluation period, glucose and creatinine levels were higher than the established thresholds but did not vary considerably from day to day. Platelet count was the only laboratory value exhibiting a notable increase from day 0 to day 7. No major changes in GCS, NIHSS, and mRS scores were observed between days 0 and 7 (Fig. 2c).

Fifteen AEs were reported, with four of nine patients experiencing at least one AE (Table 2). Two AEs (13.3%) were mild, seven (46.7%) were moderate, and six (40.0%) were severe. Only one patient experienced an AE possibly related to the MSC infusion. This patient developed a fever that resolved within 6 days with acetaminophen; no source could be identified on infectious disease workup. Most AEs (10 [66.7%]) were determined to be unrelated to MSC infusion. Patient 9 died 7 days after discharge; however, the cause of death was unrelated to the MSC infusion as determined by an independent medical examiner.

### Exploratory Imaging Volumetric Model Analysis with Clinical Characteristics

**Fig. 3:**
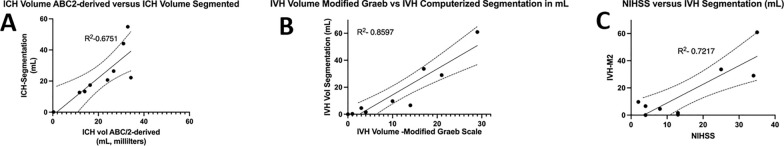
Imaging comparisons. **a**, Linear regression of neuroimaging ICH vol by model 1 ABC/2 derived method versus vol model 2 (segmentation). **b**, IVH modified Graeb by model 1 and quantitative segmentation model 2 method. **c**, Hemorrhage vol and NIHSS score compared with IVH vol by segmentation. ICH, intracerebral hemorrhage, IVH, intraventricular hemorrhage, M2, model 2, NIHSS, National Institutes of Health Stroke Scale, vol, volume

Both ABC/2 model 1 and model 2 segmentation methods showed good correlation by linear regression for ICH and IVH volumes (Fig. 3a, b). Model 1 ABC/2 derived semiquantitative methods, and modified Graeb IVH methods took less time to complete (e.g., 2–5 min) compared with model 2 segmentation volumetric methods (e.g., 15–20 min per CT). Interestingly, comparison of the NIHSS score to the IVH volumetric M2 method showed a linear correlation (*R*^*2*^ = 0.7217), and there appeared to be clustering of NIHSS scores when IVH was less than 20 mL or greater than 20 mL (Fig. 3c). PHE analysis revealed a slight negative relationship between MSC injection dosage and PHE volume.

### Analysis of Biomarkers in Blood Plasma

**Fig. 4:**
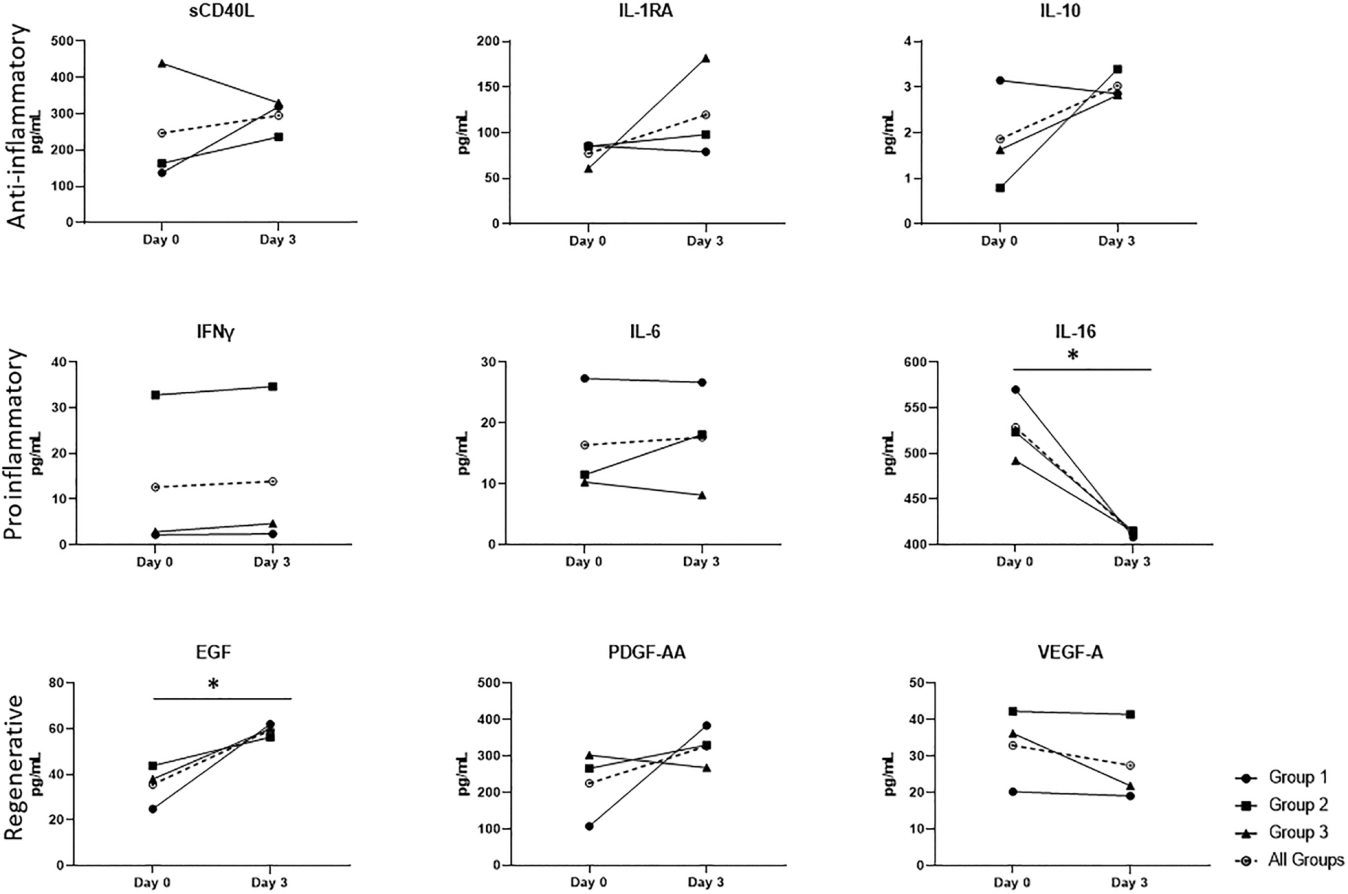
Analysis of anti-inflammatory, proinflammatory, and regenerative cytokines in the plasma of patients with ICH on day 0 before infusion and on day 3 post infusion. ICH, intracerebral hemorrhage, IVH, intraventricular hemorrhage, NIHSS, National Institutes of Health Stroke Scale, vol, volume

Blood plasma was analyzed for presence and concentration of key cytokines and chemokines on days 0 and 3 (Fig. 4). Cytokines evaluated include anti-inflammatory mediators such as soluble CD40 ligand, interleukin 1 receptor antagonist, and interleukin 10; proinflammatory cytokines, such as interferon-γ, IL-6, and interleukin 16 (IL-16); and regenerative epidermal growth factor (EGF), platelet-derived growth factor AA, and vascular endothelial growth factor A. Overall, the concentration of anti-inflammatory cytokines increased from day 0 to day 3. The uniform response pattern noted for anti-inflammatory cytokines was not observed for proinflammatory or regenerative cytokines; however, there was a decrease in proinflammatory IL-16 and increase in regenerative EGF from day 0 to day 3.

## Discussion

We describe preliminary safety and tolerability data for BM-MSC used in patients with acute ICH in a prospective, single-arm, dose-escalation clinical trial. Based on our preclinical work [24, 25, 37], we hypothesized BM-MSC may provide a pleiotropic benefit for patients with ICH with both primary-injury and secondary brain-injury mechanisms, given increasing evidence that central nervous system and systemic inflammation exist. A multipronged approach to ICH using BM-MSC seems reasonable given that no single therapy alone has been shown to improve ICH outcomes [1]. Efforts remain focused on providing multipronged, multidisciplinary ICH care by using a systems-engineering approach [38], starting in the prehospital phase, followed by intrahospital and intensive care, posthospital rehabilitation care, and secondary ICH prevention phases.

Our preliminary results appear to support our hypothesis that MSC therapy is generally safe and tolerable when given intravenously, and overall, the data appear consistent with other preclinical MSC data [24, 29]. The preliminary results suggest MSC therapy should be tested in a larger, prospective, placebo-controlled clinical trial. A future phase I or II trial could further validate the hypothesis that BM-MSCs are safe and tolerable, and if powered appropriately, begin to assess differences in outcomes.

We acknowledge several limitations in this small pilot study, including the lack of a randomized control arm. However, we introduced and evaluated various biomarkers and neuroimaging techniques that can be translated to a larger-scale phase I or II trial to further test safety, tolerability, and whether there is signal for potential efficacy. Our preliminary biomarker analysis revealed a significant reduction in IL-16. Although the exact role of IL-16 in ICH is not defined, in patients with focal cerebral infarctions, IL-16 accumulates in the perivascular region to promote inflammation [39]. If a similar mechanism exits in ICH, our data suggest that MSCs might play a role in decreasing IL-16 expression or secretion. The neuroprotective effect of EGF is well established [40, 41], and the significant increase in EGF noted in these patients 3 days after MSC treatment aligns with this mechanism of action. Although we have noted overall trends across cytokine classes and differences for specific cytokines, the lack of a control group makes it difficult to quantify the extent of these changes mediated by MSC treatment. Ahn et al. [4] revealed potential biomarkers of interest, such as cerebrospinal fluid IL-6, that should be further investigated in future human ICH phase II trials. Furthermore, during our clinical study, we saw the literature evolve to highlight the growing importance of NLR in hemorrhagic stroke. NLR is a promising, low-cost, systemic inflammation biomarker that can be derived from the complete blood cell count on most admission blood tests, assuming a differential has been obtained. NLR can predict clinical progression for cancers and has been shown to predict worse outcomes for patients with ICH [16, 42, 43] and subarachnoid hemorrhage [17].

Another limitation of our trial is its single-center design because it relied on referring hospitals to call and request transport of their patients with ICH after being admitted at their hospital hours to days earlier. To address this, we created awareness of the trial in our stroke community and screened a large number of ICH cases at our comprehensive stroke center that mirrored the eligibility criteria derived from prior ICH trials. Early in the trial, we received feedback that the requirement of within 72 h of ICH onset was too restrictive and not practical for most hospitals to transport patients with ICH to our center. In reviewing screening logs, extending the ICH window did help recruitment, and this became especially true during 2020 when the COVID-19 pandemic began. We learned that many patients with ICH were admitted on nights and weekends and were being actively stabilized at their local hospital for 48 to 72 h, and we were frequently called after that timeframe. Because hyperdense ICH blood remains visible for up to a week or longer, we thought it was biologically plausible to extend the MSC treatment window to include up to 168 h from onset to recruit more patients. We also discovered challenges in off-hours obtaining formal informed consent, and we could not pursue Exception From Informed Consent during emergencies from our institutional review board because we did not have human safety and tolerability data [44]. We learned that future BM-MSC trials will require an onsite cell therapy laboratory ready to process these bioactive cells under stringent quality control measures and with the appropriate level of nursing and infusion safety monitoring for AE reporting. Most of these practical challenges were overcome by extending the ICH MSC time window up to 168 h. As mentioned, when the COVID-19 pandemic began, study recruitment was limited by a critical hospital census and overcapacity issues in the intensive care unit and difficulties obtaining written informed consent from surrogates who were not allowed to enter the hospital. This hospital policy was later overturned to allow one family member or legal surrogate into the patient’s room, but by the time this occurred, the trial was beyond the midpoint of enrollment. In addition, we had initially planned to include a 4th dose group in which three study participants would receive 0.5 million cells/Kg intraventricularly. However, patients in this group would need to have an EVD catheter already in place; this requirement coupled with the COVID-19 pandemic, resulted in the elimination of this intervention group.

Regarding neurosurgical intervention for ICH, we acknowledge that since we began the trial, new minimally invasive surgery (MIS) techniques have been introduced, including parafascicular and tubular retractor methods of ICH extraction. These MIS ICH extraction techniques have shown promising preliminary results compared with open craniotomy ICH evacuation methods [45]. In our trial, the neurosurgeon was allowed to choose either an MIS approach or open craniotomy and to place an EVD per standard of care when deemed appropriate for hydrocephalus. Patients 1, 5, 6, and 9 received EVDs. Patient 5, a 36-year-old woman with an initial NIHSS score of 34, had an EVD initially placed but ICP became refractory, and the neurosurgeon performed a right frontal MIS tubular retractor method of ICH removal with successful ICH evacuation [46]. This patient’s outcome exceeded our expectations based on her initial GCS and ICH scores of 7 and 3, respectively (> 70% mortality). During outpatient follow-up, her NIHSS score decreased to 2 [46]. However, given the preliminary nature of this trial, we cannot make any claims about efficacy based on a single data point. This patient’s case illustrates the need to find the right signal in the large amount of multidimensional data for a biologically complex cerebrovascular disease such as ICH.

Finally, as alluded to in the recent American Heart Association ICH 2022 guideline, a redesign of our ICH systems of care [38, 47] is needed to identify and act with greater immediacy in both prehospital and early hospital phases of care. The phrase, “time is brain,” applies to patients with ICH, too, with mathematical estimates suggesting that a 33-mL ICH equates to about 2.2 billion neurons lost, and may explain why patients with ICH have a disproportionately higher 30-day mortality and disability [1, 48]. To advance the science in ICH stroke care, we must recognize that there is a lack of an evidence-based drug therapy, and the role of neurosurgical intervention remains unclear. We must also examine emerging data that patients with ICH may have covert consciousness similar to other severe forms of brain injury from disrupted neuronal interconnections, and health care teams should be cautious in placing unnecessary or early do not resuscitate orders that could create a sense of hopelessness [49] and foster clinical nihilism [50] for future patients with ICH. The time is now to advance the science for patients with ICH by testing future therapies. Given the relatively lower epidemiologic incidence of ICH in the stroke population, we believe a future randomized, multicenter trial should consider using an adaptive Bayesian design to optimize patient selection given numerous covariates. Such a trial should include a secondary multiomics approach arm to investigate the potential role of NLR, cytokines, and newer machine learning neuroimaging and high-dimensional data analysis techniques that may be able to find possible ICH endophenotypes responsive to new therapeutic interventions.

## Conclusions

We report the preliminary safety and tolerability data of three doses of BM-MSC in patients with acute ICH. The most common AE reported was fever, which was treatable with acetaminophen. Further large, randomized trials are warranted to test the hypothesis that BM-MSC infusion is a safe, tolerable, and potentially efficacious therapy to reduce the deleterious inflammatory cascade, improve outcomes in conjunction with newer MIS neurosurgical interventions, and possibly reduce PHE around the ICH.

### Supplementary Information

Below is the link to the electronic supplementary material.

Supplementary file1 (TIF 374 KB)

Supplementary file2 (DOCX 17 KB)

Supplementary file3 (DOCX 14 KB)

## Clinical Trial Registration

ClinicalTrials.gov NCT03371329.
