# Supplementary figures and images for: Mesenchymal Stem Cell Therapy in Acute Intracerebral Hemorrhage: A Dose-Escalation Safety and Tolerability Trial
Source: Neurocrit Care. 2023 Dec 19;41(1):59–69. doi: 10.1007/s12028-023-01897-w (PMC11335835; doi:10.1007/s12028-023-01897-w)

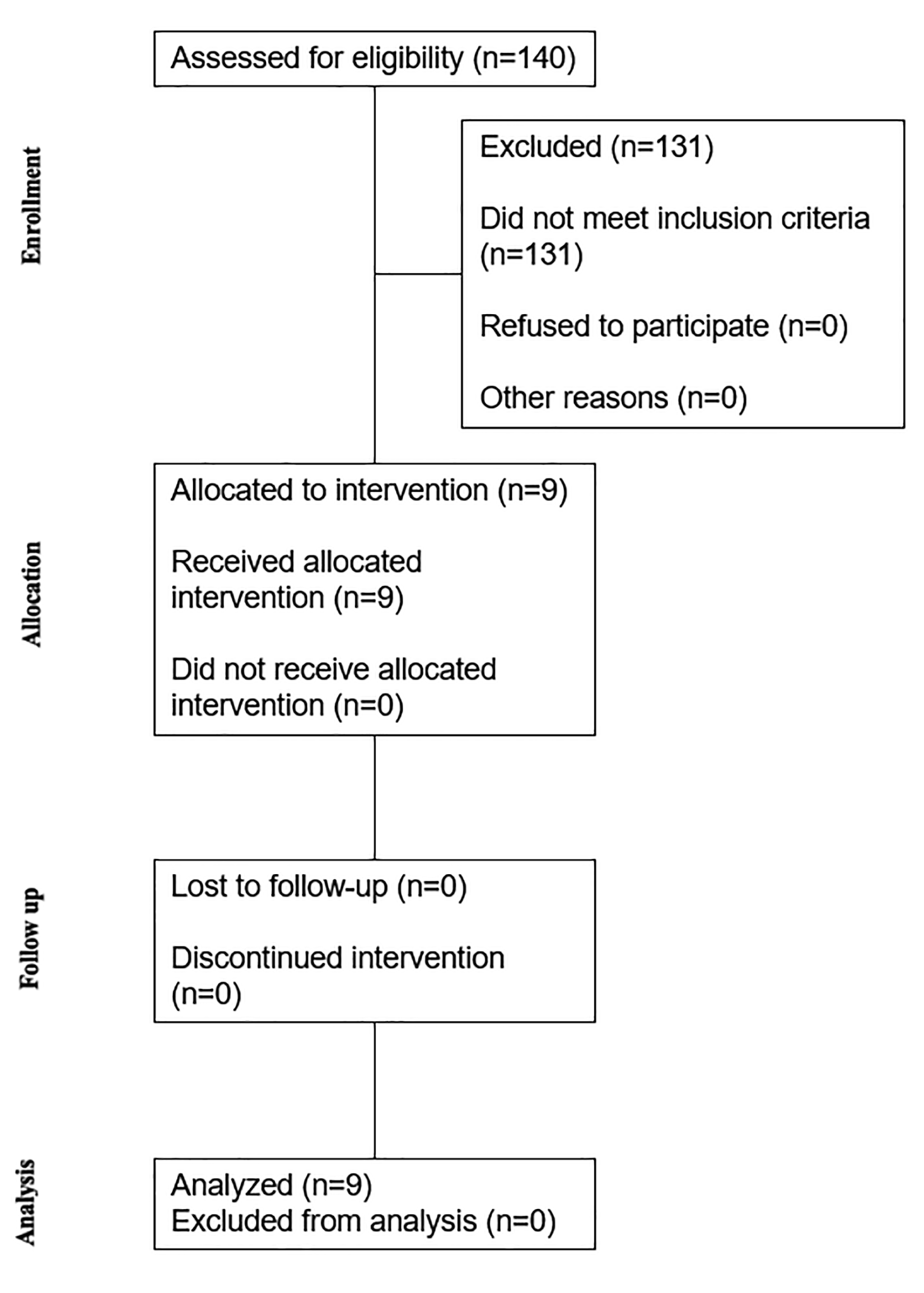

Supplement: Supplementary file 1 — Supplementary file1 (TIF 374 KB) [file 12028_2023_1897_MOESM1_ESM.tif]
