# Supplementary material for: Mesenchymal Stem Cell Therapy in Acute Intracerebral Hemorrhage: A Dose-Escalation Safety and Tolerability Trial
Source: Neurocrit Care. 2023 Dec 19;41(1):59–69. doi: 10.1007/s12028-023-01897-w (PMC11335835; doi:10.1007/s12028-023-01897-w)
Supplement: Supplementary file 2 — Supplementary file2 (DOCX 17 KB) [file 12028_2023_1897_MOESM2_ESM.docx]

**Supplemental Table 1.** Inclusion and Exclusion Criteria for Enrolled Patients^28^

| Inclusion Criteria:   1. Age: 18 years or older 2. Sex: Male or female 3. Acute spontaneous supratentorial ICH documented by head CT with ICH score of 1-4 4. Stable ICH hematoma volume ≤60mL by 24-72 h measured with ABC/2 method^27^ 5. Ability to be enrolled within 168 h of onset of stroke symptoms 6. Ability to provide written personal or surrogate consent. Only patients who have signed the ICF will be enrolled. The ICF will include elements required by Mayo IRB and FDA in US 21CFR50. 7. Adequate renal function: creatinine <1.5g/dL 8. Available for all specified assessments at the study site through the completion of the study 9. Ability to provide written ICF and authorization for use of and disclosure of protected health information. 10. MIS ICH evacuation (burr-hole craniotomy or similar) performed with either the NICO Brain path stereotactic system or the Penumbra aspiration system if done within the first 72 h of ICH onset, or symptomatic neurologic deterioration from ICH mass effect within the first 72 h of ICH onset 11. Placement of an external ventricular catheter is permissible and considered MIS, since placed through a frontal burr-hole for symptomatic hydrocephalus and cerebrospinal fluid diversion |
| --- |
| Exclusion Criteria:   1. Deep coma defined as ≤5 on the Glasgow Coma Scale 2. Open craniotomy neurosurgical evacuation of ICH 3. Secondary ICH related to aneurysm, arteriovenous malformation, trauma, brain tumor, or oral anticoagulants other than warfarin. Apixaban and rivaroxaban-related ICH will be permitted if reversed with FDA-approved coagulant factor Xa per Mayo Pharmacy protocol, as well as dabigatran reversal with idarucizumab. However, use of other novel anticoagulants (eg, edoxaban) without FDA-approved antidote is grounds for exclusion 4. Pregnancy or breast-feeding 5. Pre-existing disability defined as a prestroke modified Rankin scale >2a or clinically significant illness with manifestations of significant organ dysfunction that the investigators believe would render the patient unlikely to tolerate the MSC infusion or complete the study 6. Evidence or history of malignancy in the last 5 years (history of basal cell carcinoma is permitted) 7. Evidence of liver dysfunction; liver profile showing alkaline phosphatase >115 U/L, total bilirubin >1.0 mg/dL, alanine aminotransferase >55 U/L, and aspartate aminotransferase >48 U/L 8. Evidence of significant cardiac dysfunction 9. Septicemia with high fever and hemodynamic instability 10. Experimental therapy (drug or biologic) for any indication within 3 mo of study enrollment |

Abbreviations: CT, computed tomography; FDA, US Food and Drug Administration; ICF, informed consent form; ICH, intracerebral hemorrhage; IRB, institutional review board; MIS, minimally invasive surgery.
