# Supplementary material for: Mesenchymal Stem Cell Therapy in Acute Intracerebral Hemorrhage: A Dose-Escalation Safety and Tolerability Trial
Source: Neurocrit Care. 2023 Dec 19;41(1):59–69. doi: 10.1007/s12028-023-01897-w (PMC11335835; doi:10.1007/s12028-023-01897-w)
Supplement: Supplementary file 3 — Supplementary file3 (DOCX 14 KB) [file 12028_2023_1897_MOESM3_ESM.docx]

**Supplemental Table 2.** Characteristics of MSC Infusion By Patient

| **Patient** | **Weight, kg** | **Prescribed cell dose, million/kg** | **Preinfusion viability, %** | **Gram stain** | **Culture** |
| --- | --- | --- | --- | --- | --- |
| 1 | 78.9 | 0.5 | 91.5 | NOS | Negative |
| 2 | 96.2 | 0.5 | 83.5 | NOS | Negative |
| 3 | 87.0 | 0.5 | 73.2 | NOS | Negative |
| 4 | 56.1 | 1.0 | 83.3 | NOS | Negative |
| 5 | 97.8 | 1.0 | 81.8 | NOS | Negative |
| 6 | 121.0 | 1.0 | 78.1 | NOS | Negative |
| 7 | 74.3 | 2.0 | 71.1 | NOS | Negative |
| 8 | 70.0 | 2.0 | 71.4 | NOS | Negative |
| 9 | 94.2 | 2.0 | 70.0 | NOS | Negative |

Abbreviations: MSC, mesenchymal stem/stromal cell; NOS, no organism seen.
